# Optimal cut-off of obesity indices to predict cardiovascular disease risk factors and metabolic syndrome among adults in Northeast China

**DOI:** 10.1186/s12889-016-3694-5

**Published:** 2016-10-13

**Authors:** Jianxing Yu, Yuchun Tao, Yuhui Tao, Sen Yang, Yaqin Yu, Bo Li, Lina Jin

**Affiliations:** 1 Epidemiology and Biostatistics, School of Public Health, Jilin University, NO. 1163 Xinmin Street, Changchun, 130021 Jilin China; 2 Department of Immunization Program, Changchun Center for Disease Control and Prevention, Changchun, 130021 Jilin China

**Keywords:** ROC, Optimal cut-off, Obesity indices, Cardiovascular diseases, Metabolic syndrome

## Abstract

**Background:**

CVD risk factors (hypertension, dyslipidemia and diabetes) and MetS are closely related to obesity. The selection of an optimal cut-off for various obesity indices is particularly important to predict CVD risk factors and MetS.

**Methods:**

Sixteen thousand seven hundred sixty-six participants aged 18–79 were recruited in Jilin Province in 2012. Five obesity indices, including BMI, WC, WHR, WHtR and BAI were investigated. ROC analyses were used to evaluate the predictive ability and determine the optimal cut-off values of the obesity indices for CVD risk factors and MetS.

**Results:**

BMI had the highest adjusted ORs, and the adjusted ORs for hypertension, dyslipidemia, diabetes and MetS were 1.19 (95 % CI, 1.17 to 1.20), 1.20 (95 % CI, 1.19 to 1.22), 1.12 (95 % CI, 1.10 to 1.13), and 1.40 (95 % CI, 1.38 to 1.41), respectively. However, BMI did not always have the largest adjusted AUROC. In general, the young age group (18 ~ 44) had higher ORs and AUROCs for CVD risk factors and MetS than those of the other age groups. In addition, the optimal cut-off values for WC and WHR in males were relatively higher than those in females, whereas the BAI in males was comparatively lower than that in females.

**Conclusions:**

The appropriate obesity index, with the corresponding optimal cut-off values, should be selected in different research studies and populations. Generally, the obesity indices and their optimal cut-off values are: BMI (24 kg/m^2^), WC (male: 85 cm; female: 80 cm), WHR (male: 0.88; female: 0.85), WHtR (0.50), and BAI (male: 25 cm; female: 30 cm). Moreover, WC is superior to other obesity indices in predicting CVD risk factors and MetS in males, whereas, WHtR is superior to other obesity indices in predicting CVD risk factors and MetS in females.

**Electronic supplementary material:**

The online version of this article (doi:10.1186/s12889-016-3694-5) contains supplementary material, which is available to authorized users.

## Background

With economic development and the improvement of living conditions, the prevalence of obesity is increasing dramatically in China [1, 2]. A number of studies have demonstrated that obesity is associated with hypertension, dyslipidemia, diabetes and MetS [3–5], and hypertension, dyslipidemia and diabetes are considered risk factors for CVD [6, 7].

To evaluate obesity, many indices have been proposed, including BMI, WC, WHR, WHtR and BAI. Generally, BMI is one of the most commonly used indices for obesity, which approximates body mass using a mathematical ratio of weight and height [8]. WC is the central diagnostic index of obesity and only considers abdominal obesity [9]. WHR and WHtR are indices for evaluating fat distribution using WC compared to HC or height [10, 11]. Finally, BAI is an index to measure the amount of body fat that uses HC compared to height [12]. Obviously, other indices may be used to measure obesity, but we do not consider all of them here.

Some studies indicated that WC or WHtR might be better predictors for CVD risk factors or MetS in Korean/Chinese populations [9, 13], whereas, Mbanya et al. noted that WC was the best predictor in Cameroonians [14]. Moreover, Bergman et al. found that BAI was a better predictor for African-Americans and Mexican-Americans [12], However, Lam et al. proposed that BAI is not likely to be better than BMI and does not apply to Asians [11]. Therefore, selection of the proper obesity index for specific research and study populations was a challenge.

In our study, the predictive ability and the optimal cut-off values of five obesity indices (BMI, WC, WHR, WHtR and BAI) for CVD risk factors and MetS are comprehensively investigated. Data from 16,766 participants aged 18–79 in Jilin Province were used to evaluate the obesity indices. Jilin is in central northeast China and has an annual average temperature 4.8 °C (latitude 40° ~ 46°, longitude 121° ~ 131°) [15]. Therefore, the results can be instructive and meaningful for studies related to obesity in northeast China. WC and WHtR are superior to other obesity indices in predicting CVD risk factors and MetS in our study, with optimal cut-off values of WC and WHtR of 85 (male)/80 (female) and 0.5, respectively.

## Methods

### Study population

A large-scale cross-sectional survey was implemented in Jilin Province in 2012. A total of 16,766 participants who had lived in Jilin Province for more than 6 months and were 18–79 years old were selected through multistage stratified random cluster sampling (see details in Part 1 of the Additional file 1).

### Data measurement

Height, weight, WC and HC were measured according to a standardized protocol and techniques, with the participants wearing light clothing but no shoes. Blood pressure was measured by trained professionals using a mercury sphygmomanometer. After an overnight fast, FBG and serum lipids were measured before breakfast using a Bai Ankang fingertip blood glucose monitor (Bayer, Leverkusen, Germany) and a MODULE P800 biochemical analysis machine (Roche Co., Ltd., Shanghai, China), respectively (see details in Part 2 of the Additional file 1).

The various obesity indices were calculated as follows:

$$ \mathrm{B}\mathrm{M}\mathrm{I}=\frac{\mathrm{weight}\left(\mathrm{kg}\right)}{\mathrm{heigh}{\mathrm{t}}^2\left(\mathrm{m}\right)},\mathrm{W}\mathrm{H}\mathrm{R}=\kern0.5em \frac{\mathrm{WC}\left(\mathrm{cm}\right)}{\mathrm{HC}\left(\mathrm{cm}\right)},\mathrm{WH}\mathrm{t}\mathrm{R}=\kern0.5em \frac{\mathrm{WC}\left(\mathrm{cm}\right)}{\mathrm{heigh}\mathrm{t}\left(\mathrm{cm}\right)},\mathrm{B}\mathrm{A}\mathrm{I}=\kern0.5em \frac{\mathrm{HC}\left(\mathrm{cm}\right)}{\mathrm{heigh}{\mathrm{t}}^{1.5}\left(\mathrm{m}\right)}\hbox{-} 18 $$

### Assessment criteria

CVD risk factors refer to hypertension, dyslipidemia and diabetes in our study. Hypertension was defined as resting SBP ≥140 mmHg and/or DBP ≥ 90 mmHg and/or by the use of antihypertensive medication in the past two weeks [16]. Dyslipidemia was defined as use of lipid-lowering drugs or having one or more of the following: TG ≥ 1.7 mmol/L, TC ≥ 5.2 mmol/L, HDL-C < 1.0 mmol/L and LDL-C ≥ 3.4 mmol/L [17]. Diabetes was defined as the use of hypoglycemic agents or a self-reported history of diabetes or FBG of 7.0 mmol/L or more [18]. MetS [19, 20] was defined as three or more of the following conditions clustered in one subject: a) WC ≥ 85 cm for males or ≥ 80 cm for females; b) TG ≥ 1.7 mmol/L or ongoing hypertriglyceridemia treatment; c) HDL-C < 1.00 mmol/L for males or < 1.30 mmol/L for females, or ongoing treatment; d) SBP ≥ 130 mmHg and DBP ≥ 85 mmHg, or ongoing antihypertensive drug therapy; and e) FBG ≥ 5.6 mmol/L or ongoing anti-diabetic drug treatment.

### Statistical analyses

The continuous variables were expressed as the means ± standard deviations (SD) and compared using the *t* test. The categorical variables were expressed as counts or percentages and compared using the Rao-Scott-χ^2^ test. ROC analyses were used to compare the predictive ability and determine the optimal cut-off values of the various obesity indices for CVD risk factors and MetS [21]. The value that led to the maximum Youden index (SEN + SPE −1) [22] was taken as the optimal cut-off value, and the AUROC was the index of the predictive ability. Logistic regression models were used to calculate the ORs and to evaluate the obesity indices. All statistical analyses were performed using IBM SPSS 20.0. (SPSS Inc., New York, NY, USA) Statistical significance was set at a *P* value < 0.05.

## Results

The characteristics of the participants are shown in Table 1. Females had a higher age, TC, LDL-C and HDL-C than males (*P* < 0.05), but other anthropometric indices were significantly higher in males than those in females (*P* < 0.01). The prevalence of hypertension, dyslipidemia, diabetes, and MetS differed significantly by gender and were higher in males than in females (*P* < 0.05).

**Table 1:** Descriptive characteristics of the participants by gender

Variable	All	Male	Female	*t/*χ^*2*^	*P* value
	(*n* = 16766)	(*n* = 7697)	(*n* = 9069)		
Age(years)	47.80 ± 13.18	47.00 ± 13.74	48.47 ± 12.66	−7.20	<0.001
Height(cm)	162.84 ± 8.62	169.23 ± 6.59	157.41 ± 6.04	120.19	<0.001
Weight(kg)	64.49 ± 11.84	69.80 ± 11.91	59.98 ± 9.72	57.84	<0.001
WC(cm)	82.39 ± 10.52	84.70 ± 10.44	80.44 ± 10.19	26.58	<0.001
HC(cm)	95.08 ± 7.23	95.58 ± 7.2	94.66 ± 7.23	8.23	<0.001
SBP(mmHg)	131.35 ± 21.33	134.46 ± 19.75	128.71 ± 22.24	17.74	<0.001
DBP(mmHg)	80.01 ± 11.74	82.33 ± 11.73	78.04 ± 11.39	23.89	<0.001
TC(mmol/L)	4.90 ± 1.08	4.88 ± 1.06	4.92 ± 1.10	−2.50	0.012
TG(mmol/L)	1.96 ± 1.80	2.17 ± 2.09	1.79 ± 1.49	13.51	<0.001
LDL-C(mmol/L)	2.94 ± 0.89	2.89 ± 0.86	2.98 ± 0.92	−6.25	<0.001
HDL-C(mmol/L)	1.39 ± 0.39	1.35 ± 0.41	1.42 ± 0.36	−11.89	<0.001
FBG (mmol/L)	5.38 ± 1.66	5.52 ± 1.68	5.27 ± 1.64	9.94	<0.001
Hypertension	6249(37.27 %)	3162(41.08 %)	3087(34.04 %)	88.31	<0.001
Dyslipidemia	6679(39.76 %)	3410(44.30 %)	3269(36.05 %)	118.44	<0.001
Diabetes	1688(10.07 %)	820(10.65 %)	868(9.57 %)	5.39	0.02
MetS	5535(33.01 %)	2638(34.27 %)	2897(31.94 %)	10.21	0.001

For an overview of each obesity index, Table 2 presents the adjusted ORs and AUROCs (adjusted for gender and age). In general, BMI had the highest adjusted ORs for CVD risk factors and MetS, but it did not always have the largest adjusted AUROC. BMI, WC and WHtR had the optimal adjusted AUROC for hypertension, whereas WC, WHR and BMI had the largest adjusted AUROC for dyslipidemia, diabetes and MetS, respectively. Moreover, BAI did not have a better adjusted OR or AUROC for any CVD risk factor or MetS in our study.

**Table 2:** Adjusted ORs and adjusted AUROC for obesity indices in relation to CVD risk factors and MetS

	Hypertension		Dyslipidemia		Diabetes		MetS	
	Adjusted OR(95 % CI)	AUROC (95 % CI)	Adjusted OR(95 % CI)	AUROC (95 % CI)	Adjusted OR(95 % CI)	AUROC (95 % CI)	Adjusted OR(95 % CI)	AUROC (95 % CI)
BMI	**1.19(1.17,1.20)**	**0.77(0.76,0.78)**	**1.20(1.19,1.22)**	0.71(0.70,0.72)	**1.12(1.10,1.13)**	0.73(0.72,0.74)	**1.40(1.38,1.41)**	**0.81(0.80,0.81)**
WC	1.06(1.06,1.07)	**0.77(0.76,0.78)**	1.08(1.07,1.08)	**0.73(0.72,0.73)**	1.05(1.05,1.06)	0.74(0.73,0.75)	1.15(1.14,1.16)	0.78(0.77,0.79)
WHR	1.08(1.07,1.09)	0.76(0.75,0.76)	1.12(1.11,1.12)	0.71(0.71,0.72)	1.08(1.07,1.09)	**0.75(0.73,0.76)**	1.19(1.18,1.20)	0.78(0.78,0.79)
WHtR	1.11(1.10,1.11)	**0.77(0.76,0.78)**	1.13(1.12,1.14)	0.72(0.71,0.73)	1.09(1.08,1.10)	0.74(0.73,0.75)	1.25(1.24,1.26)	0.79(0.78,0.80)
BAI	1.13(1.12,1.14)	0.75(0.74,0.76)	1.13(1.12,1.14)	0.66(0.65,0.67)	1.07(1.07,1.08)	0.71(0.70,0.72)	1.23(1.21,1.24)	0.75(0.74,0.76)

The OR and AUROC were adjusted for gender and age

Then, the detailed performance of 5 obesity indices associated with CVD risk factors and MetS was investigated. For females (Table 3), the ORs and AUROCs of the obesity indices for CVD risk factors and MetS were the largest in the 18 ~ 44 age group, followed by the 45 ~ 64 group. Thus, obesity in the younger age groups was at a higher risk for CVD risk factors and MetS (higher ORs), and it had better predictive ability for CVD risk factors and MetS as well (larger AUROC). Further, the AUROC for males had a similar tendency and characteristics as that of females (see Additional file 1: Table S3).

**Table 3:** ORs and AUROCs for the obesity indices in relation to CVD risk factors and MetS in females by age group

	18 ~ 44		45 ~ 64		65 ~ 79	
	OR	AUROC	OR	AUROC	OR	AUROC
Hypertension						
BMI	1.23(1.20,1.26)	0.70(0.68,0.72)	1.17(1.15,1.19)	0.64(0.62,0.66)	1.11(1.07,1.16)	0.66(0.62,0.69)
WC	1.09(1.07,1.10)	0.70(0.68,0.72)	1.06(1.05,1.07)	0.64(0.62,0.66)	1.04(1.02,1.06)	0.65(0.61,0.69)
WHR	1.09(1.08,1.11)	0.69(0.67,0.71)	1.08(1.07,1.09)	0.63(0.61,0.65)	1.04(1.02,1.06)	0.64(0.60,0.68)
WHtR	1.14(1.12,1.16)	0.70(0.68,0.72)	1.10(1.09,1.12)	0.64(0.62,0.66)	1.06(1.04,1.09)	0.67(0.63,0.70)
BAI	1.17(1.14,1.20)	0.66(0.64,0.68)	1.12(1.10,1.14)	0.60(0.58,0.62)	1.07(1.03,1.11)	0.64(0.60,0.68)
Dyslipidemia						
BMI	1.18(1.16,1.21)	0.74(0.72,0.76)	1.15(1.12,1.17)	0.71(0.69,0.72)	1.08(1.05,1.12)	0.69(0.65,0.72)
WC	1.07(1.06,1.08)	0.75(0.73,0.76)	1.06(1.06,1.07)	0.72(0.70,0.73)	1.05(1.03,1.06)	0.68(0.65,0.72)
WHR	1.10(1.08,1.11)	0.74(0.72,0.76)	1.10(1.08,1.11)	0.70(0.68,0.71)	1.06(1.04,1.09)	0.67(0.63,0.71)
WHtR	1.12(1.10,1.13)	0.75(0.73,0.77)	1.10(1.09,1.11)	0.71(0.70,0.73)	1.06(1.04,1.08)	0.68(0.64,0.71)
BAI	1.12(1.10,1.15)	0.68(0.67,0.70)	1.09(1.07,1.10)	0.63(0.62,0.65)	1.03(1.00,1.06)	0.60(0.56,0.64)
Diabetes						
BMI	1.17(1.12,1.22)	0.65(0.61,0.70)	1.10(1.07,1.12)	0.62(0.59,0.64)	1.11(1.06,1.16)	0.62(0.57,0.67)
WC	1.08(1.06,1.10)	0.68(0.64,0.73)	1.05(1.04,1.06)	0.65(0.62,0.67)	1.04(1.02,1.06)	0.61(0.56,0.66)
WHR	1.10(1.07,1.14)	0.70(0.66,0.74)	1.10(1.08,1.11)	0.65(0.63,0.67)	1.03(1.01,1.06)	0.60(0.54,0.65)
WHtR	1.13(1.10,1.17)	0.69(0.65,0.74)	1.08(1.07,1.10)	0.64(0.61,0.66)	1.07(1.04,1.10)	0.61(0.56,0.66)
BAI	1.10(1.05,1.16)	0.63(0.58,0.67)	1.06(1.03,1.08)	0.56(0.54,0.59)	1.07(1.03,1.12)	0.56(0.51,0.61)
MetS						
BMI	1.39(1.35,1.43)	0.84(0.82,0.85)	1.32(1.29,1.34)	0.80(0.78,0.81)	1.24(1.19,1.30)	0.79(0.76,0.82)
WC	1.17(1.15,1.18)	0.86(0.85,0.87)	1.13(1.12,1.14)	0.83(0.82,0.84)	1.10(1.08,1.12)	0.83(0.80,0.85)
WHR	1.18(1.16,1.20)	0.83(0.81,0.84)	1.17(1.15,1.18)	0.79(0.77,0.80)	1.11(1.08,1.13)	0.77(0.74,0.80)
WHtR	1.26(1.24,1.29)	0.85(0.84,0.86)	1.21(1.19,1.22)	0.81(0.80,0.83)	1.15(1.12,1.18)	0.81(0.78,0.84)
BAI	1.27(1.24,1.3)	0.75(0.73,0.77)	1.18(1.16,1.2)	0.69(0.68,0.71)	1.11(1.07,1.15)	0.69(0.65,0.73)

The detailed optimal operating points (OOPs) for BMI, WC, WHR, WHtR and BAI to predict CVD risk factors and MetS are given in Table 4, in which the OOP is the cut-off value that leads to the maximum Youden index (SEN + SPE −1) [22]. Obviously, the OOPs for different risk factors were different, so we chose a single accessible value (close to the mean of the OOPs) as the optimal cut-off value for each index. For example, the OOPs of BMI for CVD risk factors and MetS ranged from 23.24 to 24.48, so we chose 24 as the optimal cut-off value for BMI, whereas the OOPs of WC ranged from 84.13 to 85.74 for males and 79.32 to 81.58 for females, so we chose 85 and 80 as the optimal WC cut-off values. Similarly, the optimal cut-off value for WHR was 0.88 and 0.85, for WHtR was 0.5, and for BAI was 25 and 30, respectively. In addition, the optimal cut-off values of BMI and WHtR were the same in both genders, whereas the optimal cut-off values of WC and WHR in males were relatively higher than those in females, but the opposite occurred for BAI. Generally, most of the optimal index cut-off values were the same as or similar to other studies in literature [10, 11, 13, 23].

**Table 4:** Optimal operating points of the obesity indices for predicting CVD risk factors and MetS

	BMI			WC			WHR			WHtR			BAI		
	OOP (kg/m^2^)	SEN (%)	SPE (%)	OOP (cm)	SEN (%)	SPE (%)	OOP	SEN (%)	SPE (%)	OOP (cm/kg)	SEN (%)	SPE (%)	OOP	SEN (%)	SPE (%)
Male															
Hypertension	23.24	73.41	49.03	84.56	67.13	57.21	0.88	71.78	54.14	0.48	76.11	51.11	24.74	70.12	51.13
Dyslipidemia	23.81	72.29	61.01	84.13	70.19	64.64	0.88	72.32	59.87	0.49	74.04	60.62	24.83	69.14	53.18
Diabetes	24.46	63.72	56.18	85.74	69.13	56.32	0.89	71.14	57.43	0.50	74.28	51.21	25.11	67.79	47.81
MetS	24.48	78.10	70.47	84.92	88.62	70.71	0.88	82.31	64.76	0.51	80.54	72.04	25.10	74.63	58.42
Female															
Hypertension	23.64	71.33	56.39	80.14	71.71	63.12	0.85	71.13	60.55	0.51	71.42	65.36	29.44	71.12	56.23
Dyslipidemia	23.25	75.17	51.12	79.32	72.62	58.81	0.84	72.22	57.68	0.50	73.83	58.87	29.12	69.73	51.22
Diabetes	24.47	63.42	58.17	81.58	71.88	58.59	0.86	74.83	61.42	0.52	77.34	56.69	30.35	59.14	58.54
MetS	24.16	77.13	66.76	79.86	90.47	66.47	0.85	79.54	65.83	0.51	84.39	68.03	29.29	77.82	55.63

Finally, we investigated the adjusted ORs and AUROC of each obesity index for CVD risk factors and MetS (Table 5) using the optimal cut-off values determined above. In general, the WC and WHtR had higher adjusted ORs and AUROCs for CVD risk factors and MetS, regardless of the small difference between genders. WC was superior to other obesity indices in predicting CVD risk factors and MetS in males, but WHtR was superior to other obesity indices in predicting CVD risk factors and MetS in females. Abnormal WC or WHtR was at a higher risk for CVD risk factors and MetS, whereas WC and WHtR were superior to other indices in predicting CVD risk factors and MetS.

**Table 5:** Adjusted ORs and AUROCs of the obesity indices associated with CVD risk factors and MetS

	Hypertension		Dyslipidemia		Diabetes		MetS	
	Adjusted OR (95 % CI)	AUROC (95 % CI)	Adjusted OR (95 % CI)	AUROC (95 % CI)	Adjusted OR (95 % CI)	AUROC (95 % CI)	Adjusted OR (95 % CI)	AUROC (95 % CI)
Male								
*A1*	2.62(2.37,2.89)	0.61(0.59,0.62)	3.97(3.61,4.37)	0.65(0.65,0.67)	2.34(1.99,2.74)	0.60(0.58,0.61)	8.93(7.94,10.04)	0.74(0.73,0.75)
*A2*	2.65(2.40,2.93)	0.62(0.61,0.63)	**4.21(3.82,4.63)**	**0.67(0.66,0.68)**	**2.81(2.36,3.35)**	**0.63(0.61,0.65)**	**15.81(13.94,17.92)**	**0.79(0.78,0.80)**
*A3*	2.47(2.23,2.73)	0.63(0.62,0.64)	4.05(3.66,4.47)	0.66(0.65,0.67)	2.75(2.34,3.22)	**0.63(0.62,0.65)**	8.78(7.77,9.92)	0.73(0.72,0.74)
*A4*	**2.70(2.45,2.99)**	**0.64(0.62,0.65)**	4.14(3.75,4.56)	**0.67(0.66,0.68)**	2.58(2.19,3.04)	**0.63(0.61,0.65)**	11.36(10.06,12.84)	0.76(0.75,0.77)
*A5*	2.14(1.94,2.36)	0.61(0.59,0.62)	2.59(2.36,2.85)	0.62(0.60,0.63)	1.64(1.40,1.92)	0.57(0.56,0.59)	3.94(3.54,4.38)	0.66(0.65,0.67)
Female								
*A1*	2.60(2.35,2.87)	0.64(0.62,0.65)	2.44(2.22,2.67)	0.63(0.62,0.64)	1.98(1.70,2.30)	0.61(0.59,0.63)	6.32(5.68,7.04)	0.72(0.71,0.70)
*A2*	**2.86(2.58,3.17)**	**0.68(0.66,0.69)**	2.71(2.46,2.98)	**0.66(0.64,0.67)**	2.53(2.13,2.99)	0.65(0.63,0.67)	**11.53(10.20,13.03)**	**0.78(0.77,0.79)**
*A3*	2.11(1.90,2.33)	0.66(0.65,0.67)	2.36(2.14,2.60)	0.65(0.64,0.66)	2.97(2.48,3.55)	**0.67(0.65,0.69)**	5.45(4.89,6.08)	0.72(0.71,0.74)
*A4*	2.82(2.53,3.14)	**0.68(0.67,0.69)**	**2.83(2.56,3.14)**	**0.66(0.65,0.67)**	**2.99(2.46,3.63)**	**0.67(0.65,0.68)**	10.75(9.40,12.30)	0.76(0.75,0.77)
*A5*	2.00(1.81,2.20)	0.63(0.62,0.64)	1.68(1.53,1.85)	0.60(0.59,0.61)	1.28(1.10,1.48)	0.58(0.56,0.60)	3.06(2.77,3.38)	0.67(0.65,0.68)

*A1:* BMI > 24 vs. ≤24 kg/m^2^, *A2:* WC > 85 vs. ≤85 (men) or WC > 80 vs. ≤80 (women), *A3:* WHR > 0.88 vs. ≤0.88 (men) or WHR > 0.85 vs. ≤0.85 (women), *A4:* WHtR >0.5 vs. ≤0.5, *A5:* BAI > 25 vs. ≤25 (men) or BAI >30 vs. ≤30 (women). The OR and AUROC were adjusted for age

## Discussion

The prevalence of hypertension, dyslipidemia, diabetes and MetS in our study were 37.27 %, 39.76 %, 10.07 % and 33.1 %, respectively, much higher than those in other studies [17]. It was believed that obesity was associated with CVD risk factors and MetS [3] and various obesity indices were used in literature [24, 25] to describe obesity. Unfortunately, no obesity index was consistently superior in predicting CVD risk factors and MetS, and the selection of an obesity index depended on the study population and other factors [11]. Thus, in this study, we investigated the proper obesity index and optimal cut-off values to predict CVD risk factors and MetS for a population in northeast China.

In this study, obesity in younger age groups was a higher risk and had better predictive ability for CVD risk factors and MetS than in older groups. It was implied that obesity might have more influence on young people. One possible reason was that the young people took part in fewer outdoor activities and had worse eating habits than the older people. Another possible reason was that other factors might have larger effects on CVD risk factors and MetS than obesity among older people. It was suggested that the younger the participant, the more effective it is to control obesity.

We investigated the performance of five obesity indices (BMI, WC, WHR, WHtR and BAI) for CVD risk factors and MetS in northeast China. A series of optimal cut-off values of each obesity index was determined in our study, which could provide an instructive suggestion in similar studies and populations. In summary, BMI, WC and WHtR had the same optimal cut-offs as other studies in China [13, 23], while the optimal cut-off value of WHR was a little higher [13], and that of BAI was a little lower than previous studies [12]. A probable reason might be the characteristics of Asians (especially Asian women), with smaller HC than Americans [26]. The higher tolerance of WHR for CVD risk factors and MetS might be due to the flexibility of fat for those in northeast China under the long duration of cold weather.

Further, WC and WHtR were superior to other obesity indices in our study, which was consistent with other studies [27–32]. Moreover, the global cut-off value of WHtR was 0.5, which implied that this criterion might be applied to people in northeast China [10]. Meanwhile, a number of meta-analyses on CVD risk factors outcomes suggested that 0.5 (WHtR) could be appropriate for different genders and age groups [24, 33]. Moreover, the WGOC (Working Group on Obesity in China) developed a cut-off value for central obesity (85.0 cm for male and 80.0 cm for female) using WC and overweight status (24 kg/m^2^) using BMI for the general Chinese population [34], which were coincident with those in our study. In addition, other studies in Asian countries reported cut-off values of WC for males and females of approximately 80–85 and 75–80, respectively [35, 36], that were similar to those in our study.

Here, we indicate the limitations of our study. First, the definition of MetS overlapped with that of WC, so the AUROC and adjusted ORs for MetS might be overestimated. Despite this, the optimal WC cut-off value was consistent with the definition of MetS, which could be viewed as evidence of the rationality of our study. Second, gender and age were adjusted for in our study; however, other confounders that might have impacts on CVD risk factors and MetS, such as physical activity, smoking, etc., were not under our consideration this time, which might have some slight effect on our results.

Finally, we investigated the adjusted ORs of each index, based on the proposed optimal cut-off values. Generally, WC and WHtR were superior to other indices (larger AUROC), and the people with abnormal WC or WHtR were at higher risk (higher ORs) for CVD risk factors and MetS. Obviously, both indices could measure central obesity to some extent. Thus, it might be implied that the distribution of fat was more important than the amount of fat in predicting the risk for CVD risk factors and MetS.

## Conclusions

The proper obesity index should be selected in different research studies and populations, with the corresponding optimal cut-off values. Generally, the obesity indices considered in our study and their optimal cut-off values are: BMI (24 kg/m^2^), WC (male: 85 cm; female: 80 cm), WHR (male: 0.88; female: 0.85), WHtR (0.50), and BAI (male: 25 cm; female: 30 cm). Moreover, WC is superior to other obesity indices in predicting CVD risk factors and MetS in males, but WHtR is superior to other obesity indices in predicting CVD risk factors and MetS in females.

## Additional file

Additional file 1: (DOCX 20 kb)
