# Supplementary material for: Optimal cut-off of obesity indices to predict cardiovascular disease risk factors and metabolic syndrome among adults in Northeast China
Source: BMC Public Health. 2016 Oct 13;16:1079. doi: 10.1186/s12889-016-3694-5 (PMC5062901; doi:10.1186/s12889-016-3694-5)
Supplement: Additional file 1: — (DOCX 20 kb) [file 12889_2016_3694_MOESM1_ESM.docx]

**Online Supplementary Material to**

**Optimal cut-off of obesity indices to predict cardiovascular disease risk factors and metabolic syndrome among adults in northeast China**

1. **Sampling Method**

Five-stage stratified random cluster sampling was used to select the samples under study. In the first stage, 32 districts/counties were identified in proportion to population, geographic location and ethnicity, from nine cities (Changchun, Jilin, Siping, Liaoyuan, Tonghua, Baishan, Songyuan, Baicheng and Yanbian). At the second stage, three or four towns (depending on the size of the district) were selected by stratified random sampling to guarantee the representativeness of each sample. In the third stage, three neighborhood committees were chosen by stratified random sampling from each of the towns previously selected. In the fourth stage, one village from each chosen neighborhood committee was selected by simple random sampling. In the final stage, cluster random sampling was used to identify individuals aged 18 to 79 years old from each of the villages selected for the study.

1. **Data Measurement**

Anthropometric measurements including height, weight, waist circumference (WC), hip circumference (HC), blood pressure, serum lipids and fasting blood sugar were taken. During the interview, weight, height, WC and HC were determined though standardized protocol and measured in light indoor light clothing without shoes. WC was measured midway between the inferior margin of the last rib and the crest of the ileum in a horizontal plan. HC was measured at the maximal protrusion of the buttocks. Weight was measured to the nearest 0.1 kg, and height, WC and HC were measured to the nearest 0.1 cm.

A mercury sphygmomanometer was used to measure the blood pressure in the sitting position after a 10-min rest period. The appearance of the first sound was used to define systolic blood pressure (SBP) and the disappearance of sound was used to define diastolic blood pressure (DBP). Two readings each of SBP and DBP were recorded, and the average of each measurement was used for data analysis. If the first two measurements differed by more than 5 mmHg, additional readings were taken.

Blood samples were obtained from the antecubital vein into anticoagulant tubes containing EDTA in the morning after an overnight fasting period. All of the collected samples were transported on dry ice at prearranged intervals to the central laboratory. Serum lipids including TC, TG, HLD-C and LDL-C, which were measured by a MODULE P800 biochemical analyses machine.

Fasting plasma glucose (FPG) levels were measured using the Bayer Bai Ankang fingertip blood glucose monitor machine by taking a small drop of blood from a finger onto a strip of paper in the morning after participants fasted for 10 or more hours overnight.

Table 3S. ORs and AUROC for the various anthropometric indices in relation to CVD risk factors and MetS in males by age groups

|  | 18~44 | |  | 45~64 | |  | 65~ | |
| --- | --- | --- | --- | --- | --- | --- | --- | --- |
|  | OR | AUROC |  | OR | AUROC |  | OR | AUROC |
| Hypertension |  |  |  |  |  |  |  |  |
| BMI | **1.20(1.18,1.23)** | **0.70(0.68,0.72)** |  | 1.17(1.14,1.19) | 0.64(0.62,0.66) |  | 1.19(1.13,1.25) | 0.66(0.62,0.69) |
| WC | **1.07(1.06,1.08)** | **0.70(0.68,0.72)** |  | 1.05(1.05,1.06) | 0.64(0.62,0.66) |  | 1.06(1.04,1.07) | 0.65(0.61,0.69) |
| WHR | **1.10(1.09,1.11)** | 0.69(0.67,0.71) |  | 1.08(1.07,1.09) | 0.63(0.61,0.65) |  | 1.08(1.05,1.10) | 0.64(0.6,0.68) |
| WHtR | **1.13(1.11,1.14)** | **0.70(0.68,0.72)** |  | 1.10(1.08,1.11) | 0.64(0.62,0.66) |  | 1.12(1.09,1.15) | 0.67(0.63,0.70) |
| BAI | **1.18(1.15,1.21)** | 0.66(0.64,0.68) |  | 1.11(1.09,1.14) | 0.60(0.58,0.62) |  | 1.18(1.12,1.24) | 0.64(0.60,0.68) |
| Dyslipidemia |  |  |  |  |  |  |  |  |
| BMI | 1.26(1.23,1.29) | 0.74(0.72,0.76) |  | **1.27(1.24,1.30)** | 0.71(0.69,0.72) |  | 1.22(1.17,1.29) | 0.69(0.65,0.72) |
| WC | **1.09(1.08,1.10)** | **0.75(0.73,0.76)** |  | **1.09(1.08,1.10)** | 0.72(0.70,0.73) |  | 1.07(1.05,1.09) | 0.68(0.65,0.72) |
| WHR | **1.15(1.13,1.16)** | 0.74(0.72,0.76) |  | 1.13(1.12,1.14) | 0.70(0.68,0.71) |  | 1.10(1.07,1.12) | 0.67(0.63,0.71) |
| WHtR | **1.17(1.15,1.19)** | **0.75(0.73,0.77)** |  | 1.16(1.14,1.18) | 0.71(0.70,0.73) |  | 1.12,1.09,1.15) | 0.68(0.64,0.71) |
| BAI | **1.21(1.19,1.24)** | 0.68(0.67,0.70) |  | 1.17(1.14,1.19) | 0.63(0.62,0.65) |  | 1.11(1.06,1.16) | 0.60(0.56,0.64) |
| Diabetes |  |  |  |  |  |  |  |  |
| BMI | **1.12(1.07,1.16)** | 0.65(0.61,0.70) |  | 1.12(1.09,1.16) | 0.62(0.59,0.64) |  | 1.10(1.05,1.16) | 0.62(0.57,0.67) |
| WC | **1.06(1.04,1.07)** | 0.68(0.64,0.73) |  | 1.05(1.04,1.06) | 0.65(0.62,0.67) |  | 1.04(1.02,1.06) | 0.61(0.56,0.66) |
| WHR | **1.10(1.07,1.12)** | **0.70(0.66,0.74)** |  | 1.09(1.07,1.10) | 0.65(0.63,0.67) |  | 1.05(1.02,1.08) | 0.60(0.54,0.65) |
| WHtR | **1.11(1.08,1.14)** | 0.69(0.65,0.74) |  | 1.09(1.07,1.10) | 0.64(0.61,0.66) |  | 1.06(1.03,1.1) | 0.61(0.56,0.66) |
| BAI | **1.11(1.06,1.16)** | 0.63(0.58,0.67) |  | 1.06(1.03,1.09) | 0.56(0.54,0.59) |  | 1.07(1.01,1.13) | 0.56(0.51,0.61) |
| MetS |  |  |  |  |  |  |  |  |
| BMI | 1.45(1.41,1.49) | 0.84(0.82,0.85) |  | **1.49(1.45,1.53)** | 0.80(0.78,0.81) |  | 1.44(1.35,1.54) | 0.79(0.76,0.82) |
| WC | 1.16(1.15,1.17) | **0.86(0.85,0.87)** |  | **1.17(1.16,1.18)** | 0.83(0.82,0.84) |  | 1.15(1.13,1.18) | 0.83(0.80,0.85) |
| WHR | **1.23(1.21,1.25)** | 0.83(0.81,0.84) |  | **1.23(1.21,1.25)** | 0.79(0.77,0.80) |  | 1.17(1.14,1.21) | 0.77(0.74,0.80) |
| WHtR | **1.29(1.26,1.31)** | 0.85(0.84,0.86) |  | **1.29(1.27,1.31)** | 0.81(0.80,0.83) |  | 1.26(1.21,1.31) | 0.81(0.78,0.84) |
| BAI | **1.33(1.29,1.37)** | 0.75(0.73,0.77) |  | 1.26(1.23,1.29) | 0.69(0.68,0.71) |  | 1.24(1.17,1.3) | 0.69(0.65,0.73) |
